# Comparison of culture and a multiplex probe PCR for identifying *Mycoplasma* species in bovine milk, semen and swab samples

**DOI:** 10.1371/journal.pone.0173422

**Published:** 2017-03-06

**Authors:** Alysia M. Parker, John K. House, Mark S. Hazelton, Katrina L. Bosward, Paul A. Sheehy

**Affiliations:** The University of Sydney, Sydney School of Veterinary Science, Faculty of Science, Camden, New South Wales Australia; The University of Melbourne, AUSTRALIA

## Abstract

*Mycoplasma* spp. are a major cause of mastitis, arthritis and pneumonia in cattle, and have been associated with reproductive disorders in cows. While culture is the traditional method of identification the use of PCR has become more common. Several investigators have developed PCR protocols to detect *M*. *bovis* in milk, yet few studies have evaluated other sample types or other important *Mycoplasma* species. Therefore the objective of this study was to develop a multiplex PCR assay to detect *M*. *bovis*, *M*. *californicum* and *M*. *bovigenitalium*, and evaluate its analytical performance against traditional culture of bovine milk, semen and swab samples. The PCR specificity was determined and the limit of detection evaluated in spiked milk, semen and swabs. The PCR was then compared to culture on 474 field samples from individual milk, bulk tank milk (BTM), semen and swab (vaginal, preputial, nose and eye) samples. Specificity analysis produced appropriate amplification for all *M*. *bovis*, *M*. *californicum* and *M*. *bovigenitalium* isolates. Amplification was not seen for any of the other Mollicutes or eubacterial isolates. The limit of detection of the PCR was best in milk, followed by semen and swabs. When all three *Mycoplasma* species were present in a sample, the limit of detection increased. When comparing culture and PCR, overall there was no significant difference in the proportion of culture and PCR positive samples. Culture could detect significantly more positive swab samples. No significant differences were identified for semen, individual milk or BTM samples. PCR identified five samples with two species present. Culture followed by 16S-23S rRNA sequencing did not enable identification of more than one species. Therefore, the superior method for identification of *M*. *bovis*, *M*. *californicum* and *M*. *bovigenitalium* may be dependent on the sample type being analysed, and whether the identification of multiple target species is required.

## Introduction

*Mycoplasma* species belong to the class Mollicutes and are characterized by their lack of cell wall, low G+C content [23–40%] and small genome size [0.58–1.4Mbp], making them the simplest and smallest self-replicating and free-living form of life [1]. Following its initial isolation in 1961 from a case of severe mastitis in the USA [2], *Mycoplasma bovis* is one of the most important mycoplasma pathogens in cattle worldwide [3]. *Mycoplasma bovis* has been demonstrated as a causative agent of mastitis and arthritis in adults [4], as well as pneumonia [5], arthritis [6] and otitis media [7] in calves. Several other *Mycoplasma* species are of interest in cattle with varying degrees of significance. *Mycoplasma californicum* appears to be the second most common cause of mycoplasma-associated disease [8] and is associated with mastitis in adults [9, 10] and arthritis and pneumonia in calves [11]. *Mycoplasma bovigenitalium* can be isolated from the reproductive tract of cows, and has been associated with vulvovaginitis and infertility [12], as well as dystocia and endometritis [13].

The traditional method of mycoplasma identification is by culture. Due to their simplicity and as such high nutritional demands, specialized and highly enriched media is required for their growth [14]. However assuming the appropriate growth media and atmospheric conditions of 37°C and 5% CO_2_ are used, the majority of *Mycoplasma* spp. are not intrinsically difficult to grow. While this method of identification is relatively cheap and simple, there are several limitations. Due to its slow rate of growth of 7–10 days [15], *Mycoplasma* spp. can be easily overgrown by other bacteria which may contaminate the sample, resulting in identification being very difficult or not possible. At the same time it is also important to keep the mycoplasma organism viable for growth. Therefore appropriate sample handling and storage is necessary, with the recovery rate of *Mycoplasma* spp. decreasing with increased time to processing. As such, samples must be stored at 4°C and cultured as soon as possible to avoid potential false negative results [16]. An extended interval from sampling to determination of results can also be an issue for producers who have submitted samples for mycoplasma culture. Due to the highly contagious nature of the pathogen and the impact it can have within a herd, it is important to receive diagnostic results quickly so that the infected animals can be removed from the herd to minimize spread [17]. Lastly, culture also allows the growth of *Acholeplasma* species which are often isolated alongside *Mycoplasma* species however are not considered to be pathogenic [18]. Differentiating *Acholeplasma* from *Mycoplasma* species of interest is very difficult by culture as they both present with‘fried egg’ colony morphology [14]. While *Acholeplasma* species can be distinguished using biochemical tests, results can be subjective and as such difficult to determine [19]. This can result in the reporting of false mycoplasma positive samples. Therefore positive cultures should be followed up with PCR to identify the species grown to ensure an accurate diagnosis.

In recent years, PCR has become a common method for *Mycoplasma* spp. diagnosis. Several Pan-mycoplasma PCRs have been developed to detect *Mycoplasma* spp. [20, 21]. Due to *M*. *bovis* being the most commonly isolated species, numerous *M*. *bovis* specific probe based PCRs have also been developed to increase specificity [22–24]. Diagnosis by PCR is a much more rapid method, with a turnaround of 1–2 days, however does often incur a higher cost compared to culture. Due to PCR identifying the DNA of the target organism, viability of the organism and as such sample handling and storage conditions are of less importance. The introduction of PCR has also allowed the successful identification of *Mycoplasma* spp. with no amplification of *Acholeplasma* spp. observed [19, 25, 26], minimizing the risk of false positive observations due to a lack of specificity.

Commonly, PCR is performed directly on the sample following DNA extraction. Given the clinical nature of mycoplasma in the dairy industry, the most common bovine sample type for *Mycoplasma* spp. diagnosis is milk. Several studies have developed effective DNA extraction protocols for milk samples, with a detection limit as low as 10^2^ cfu/mL being reported for *M*. *bovis* PCR assays [23, 27]. However, few studies have investigated extraction methods and the limit of detection of mycoplasma in other sample types, or for other important *Mycoplasma* species. Therefore the objective of this study was to develop a real-time multiplex PCR assay to detect three important *Mycoplasma* species, *M*. *bovis*, *M*. *californicum* and *M*. *bovigenitalium*, and evaluate its analytical performance with traditional culture of bovine milk, semen and swab samples.

## Material and methods

### Multiplex probe PCR

A species specific multiplex probe PCR assay modified from Clothier et al [23] and Boonyayatra et al [24] was developed and optimized targeting *M*. *bovis*, *M*. *californicum* and *M*. *bovigenitalium*. Reaction mixtures consisted of 0.5 mM of dNTPs, 5 mM of MgCl_2_, 0.5 U GoTaq polymerase, 1 μM of each primer set, 0.25 μM of each probe, 2.0 μL of 5x Buffer and 2 μL of DNA template in a final volume of 10 μL. Cycling conditions were 95°C for 60s, followed by 40 cycles of 95°C for 30s, 60°C for 30s and 72°C for 30s [28]. The assay was performed on a RotorGene^TM^ 3000 RT-PCR System Thermocycler using the green, yellow and orange channels for *M*. *bovis*, *M*. *californicum* and *M*. *bovigenitalium* respectively (QIAGEN Pty Ltd, Chadstone Centre, VIC, Australia). Acquisition of the data occurred during the 72°C extension step. Each PCR run contained a no template control (DNA-free water) and a positive control for each targeted *Mycoplasma* species including field strain *M*. *bovis* 07–249, *M*. *californicum* 08–2152 and *M*. *bovigenitalium* 12–1511. To validate DNA extractions from bovine samples, a separate ‘in-house’ developed control PCR assay targeting *Bos Taurus* mitochondrial cytochrome B gene was also used with the same reaction mixture, cycling conditions and instrument. Each PCR run contained a no template control (DNA-free water) and a positive *Bos Taurus* control from DNA extracted from bovine milk. All samples were run in triplicate reactions for the mycoplasma assay and in single reactions for the *Bos Taurus* assay, and were considered positive if a cycle threshold (Ct) <40 was achieved. Primer and probe sequences are shown in Table 1.

**Table 1:** Primer and probe sequences used for multiplex probe PCR and culture speciation [28].

Name	Sequence	Target
MbovF	5’-TCTAATTTTTTCATCATCGCTAATGC-3’	*uvrC* gene of *Mycoplasma bovis* [GenBank accession no. AF003959] [23]
MbovR	5’-TCAGGCCTTTGCTACAATGAAC-3’	
MbovP	5’-FAM-AACTGCATCATATCACATACT-BHQ-1-3’	
McalF	5’-GCACTTAGACGAAAGAGGGATT-3’	*rpoB* gene of *Mycoplasma californicum* [no accession no. provided] [24]
McalR	5’-GGATTATCATCACCTTTGGGACT-3’	
McalP	5’-CAL Fluor Orange 560-CGTGTTGGTTCGGAAGTGGTTCCAG-BHQ-1-3’	
MbvgF	5’-CTTTCTACGGAGTACAAAGCTAAT-3’	16S-23S rRNA intergenic spacer region of *Mycoplasma bovigenitalium* [no accession no. provided] [24]
MbvgR	5’-GAGAGAATTGTTCYCTCAAAACTA-3’	
MbvgP	5’-CAL Fluor Red 610- TATCGTCATGGCTTGGTTAGGTCCCA-BHQ-2-3’	
CytbF	5’-GAGGCGGATTCTCAGTAGACAAAG-3’	*Bos Taurus* Mitochondrial Cytochrom B gene (Genbank accession no. GQ358783.1)
CytbR	5’-GAGCCTGTTTCGTGGAGGAATA-3’	
CytbP	5’-CAL Fluor Orange 560 –CCCTTACCCGATTCTTCGCTTTCCA–BHQ-1-3’	
MycoF	5’- GGGGATGGATTACCTCCTTT -3’	16S-23S rRNA intergenic spacer region of *Mycoplasma* spp.*–*‘in-house’ (GenBank accession no. AY729934) (adapted from Tang et al [20])
MycoR	5’- TTCCAGACCCAGGCATC -3’	
	

### PCR analytical specificity

For determining the analytical specificity of the multiplex PCR probe assay, 29 Mollicutes and 10 other eubacterial isolates were used (Table 2). These were obtained from field samples submitted to the Livestock Veterinary Teaching and Research Unit Milk Quality Laboratory, Faculty of Veterinary Science at the University of Sydney (USYD, Camden, NSW, Australia), from the American Type Culture Collection (ATCC, Manassas, VA, USA), or from the International Organization for Mycoplasmology (IOM, Towson, MD, USA).

**Table 2:** Mollicute and other bacterial species used for testing analytical specificity of the multiplex probe PCR assay.

		Multiplex Probe PCR		
Organism	Lab ID	*M*. *bovis*	*M*. *californicum*	*M*. *bovigenitalium*
*Mycoplasma bovis*	USYD 07–249	+	-	-
*Mycoplasma bovis*	ATCC® 25523	+	-	-
*Mycoplasma bovis*	USYD 339	+	-	-
*Mycoplasma bovis*	USYD 582	+	-	-
*Mycoplasma californicum*	USYD 08–2152	-	+	-
*Mycoplasma californicum*	USYD DA13-10900	-	+	-
*Mycoplasma californicum*	USYD DA14-1.1470	-	+	-
*Mycoplasma californicum*	USYD DA14-1526	-	+	-
*Mycoplasma californicum*	USYD DA14-1554	-	+	-
*Mycoplasma bovigenitalium*	USYD 12–1511	-	-	+
*Mycoplasma bovigenitalium*	USYD DA14-3784	-	-	+
*Mycoplasma bovigenitalium*	USYD DA14-3806	-	-	+
*Mycoplasma bovigenitalium*	USYD DA14-995	-	-	+
*Mycoplasma bovirhinis*	USYD DA13-3.5005	-	-	-
*Mycoplasma bovirhinis*	USYD DA13-3.5011	-	-	-
*Mycoplasma bovoculi*	USYD DA13-8470	-	-	-
*Mycoplasma bovoculi*	USYD DA13-3.9238	-	-	-
*Acholeplasma granularum*	USYD DA14-4688	-	-	-
*Acholeplasma granularum*	USYD DA14-6019	-	-	-
*Acholeplasma granularum*	USYD DA14-6301	-	-	-
*Acholeplasma laidlawii*	USYD DA14-3.3033	-	-	-
*Mycoplasma zaradii*	USYD DA14-3.2996	-	-	-
*Mycoplasma zaradii*	USYD DA13-1.6828	-	-	-
*Mycoplasma zaradii*	USYD DA13-1.6838	-	-	-
*Mycoplasma dispar*	ATCC® 27140	-	-	-
*Mycoplasma agalactiae*	IOM PG2	-	-	-
*Mycoplasma alkalescens*	IOM D12	-	-	-
*Mycoplasma Leachii*	IOM PG50	-	-	-
*Mycoplasma mycoides subsp*. *capri*	IOM PG3	-	-	-
*Nocardia* spp.	USYD Nocardia	-	-	-
*Staphylococcus aureus*	USYD S.aureus	-	-	-
*Staphylococcus aureus*	ATCC 25923	-	-	-
*Streptococcus agalactiae*	USYD Strep ag	-	-	-
*Streptococcus uberis*	USYD Strep ub	-	-	-
*Streptococcus dysgalactiae*	USYD Strep dysgalactiae 100	-	-	-
*Enterrococcus faecalis*	USYD E.faecalis	-	-	-
*Escherichia coli*	USYD E.coli	-	-	-
*Corynebacterium* spp.	USYD Corynebacterium 931	-	-	-
*Klebsiella* spp.	USYD Klebsiella	-	-	-

### DNA extraction

For DNA extractions on swab samples, the swab was removed from its Amies transport medium (FL Medical FL26068) and the tip cut off into 400 μL of sterile PBS in a 1.5 mL Eppendorf tube using heat sterilized forceps. Following thorough vortexing, 200 μL of the PBS solution was transferred into a fresh 1.5 mL Eppendorf tube from which the DNA extraction process was continued using the DNeasy® Blood and Tissue kit (QIAGEN Pty Ltd, Chadstone Centre, VIC, Australia) following manufacturer’s instructions for Purification of Total DNA from Animal Tissues (Spin-Column Protocol).

For DNA extractions on semen, 200 μL of semen was combined with 200 μL of 2% Triton X 100 (Sigma Aldrich 23472–9) in TE Buffer (pH8) (Amresco E112). The sample was thoroughly vortexed followed by centrifugation at 13,000 x g for 5 min and discarding of the supernatant. The DNA extraction process was then continued on the remaining pellet using the DNeasy® Blood and Tissue kit (QIAGEN Pty Ltd, Chadstone Centre, VIC, Australia) following manufacturer’s instructions for Purification of Total DNA from Animal Tissues (Spin-Column Protocol), with centrifuge times increased to 3 min.

For DNA extractions on milk samples, 1 mL of milk was centrifuged at 13,000 x g for 5 min followed by the removal of fat and supernatant. The remaining pellet was resuspended in 90 μL of Buffer ATL (QIAGEN Pty Ltd, Chadstone Centre, VIC, Australia) and 10 μL of Proteinase K (QIAGEN Pty Ltd) and incubated at 56°C for 1–3 hrs with occasional vortexing. The DNA extraction process was then continued using the BioSprint® 96 One-For-All Vet kit (QIAGEN Pty Ltd) following manufacturer’s instructions for purification of viral nucleic acids and bacterial DNA from animal tissue homogenates, serum, plasma, other body fluids, swabs and washes. Each extraction plate included a blank containing sterile PBS which was run on the PCR as extraction blanks.

### PCR limit of detection

The limit of detection of the multiplex PCR was determined for swabs, semen and milk sample types. This was first done with a single target *Mycoplasma* species present per sample, and then with all three target species present per sample. Broth cultures from control field isolates *M*. *bovis* 07–249, *M*. *californicum* 08–2152 and *M*. *bovigenitalium* 12–1511 were used for spiking samples (data not shown). The highest concentration grown in broth for each species was used for spiking samples for both a single target species present per sample, and with all three target species present per sample. For each dilution, three extractions were performed to give three extraction series which including a negative control (non-spiked sample). Each extraction series was evaluated on a separate PCR run (between-run precision). Each extraction was evaluated in three replicates (within-run precision). This gave a total of nine replicates across three PCR runs for each dilution. The limit of detection was determined as the lowest concentration when nine out of the nine replicates across three PCR runs were positive for a given dilution.

For swabs, broth culture was spiked into sterile PBS and a 10 fold serial dilution series performed with sterile PBS. For each concentration, a swab (FL Medical FL26068) was removed from its casing, swirled in the spiked PBS, and then inserted into its Amies transport medium. For semen, broth culture was spiked into pooled semen from bulls which were culture negative for mycoplasma, and a 10 fold serial dilution series performed with the semen. For milk, broth culture was spiked into a bulk tank milk (BTM) sample which was culture negative for mycoplasma, and a 10-fold serial dilution series performed with the milk. DNA extractions were performed as previously described.

### Mycoplasma culturing

All bovine field samples were inoculated onto Mycoplasma agar [Mycoplasma agar base (Oxoid CM0401); Milli-Q water; 0.2% w/v calf thymus DNA (Sigma D1501); Mycoplasma Selective Supplement G (Oxoid SR0059C); prepared by Elizabeth Macarthur Agricultural Institute (EMAI); NSW Department of Primary Industries, NSW, Australia] and incubated at 37°C in candle jars in elevated CO_2_ levels for 5 to 10 days. Following positive mycoplasma growth, several colonies from each sample were selected and placed in PBS for speciation by an ‘in house’ developed universal *Mycoplasma* spp. conventional PCR assay modified from Tang et al [20]. Reaction mixtures contained 0.25 mM dNTPs, 2.5 mM MgCl_2_, 1.5 U of GoTaq, 0.25 μM of each primer (Table 1), 8 μL of 5x Buffer and 5 μL DNA template in a final volume of 40 μL. Cycling conditions were 94°C for 5 min, followed by 35 cycles of 94°C for 30s, 55°C for 30s, 72°C for 1 min, and a final extension of 72°C for 5 min [28]. The assay was performed on a Bio-Rad-T100 Thermocycler (Bio-Rad Laboratories Pty Ltd, Gladesville, NSW, Australia). The PCR products from this assay were then speciated via Sanger Sequencing (Australian Genome Research Facility Ltd, Sydney, NSW, Australia).

### Bovine field samples

A set of 474 field samples from bovine sources submitted to the Livestock Veterinary Teaching and Research Unit Milk Quality Laboratory, Faculty of Veterinary Science at the University of Sydney (USYD), were selected for analysis. All animal sample collection was approved by The University of Sydney Animal Ethics Committee (protocol number 2013/6046). All samples were cultured and speciated for mycoplasma upon arrival as previously described, followed by freezing of the samples at -20°C. Samples were stored at -20°C for a range of 5 days to 3 years prior to DNA extraction and PCR analysis (S4 File). To validate the PCR against culture, the following sample types were chosen. All culture positive samples chosen had been previously speciated as *M*. *bovis*, *M*. *californicum* or *M*. *bovigenitalium* as previously described.

- Swab samples (n = 95) including vaginal, preputial, nose and eye: 48 culture negative and 47 culture positive samples
- Semen samples (n = 44): 22 culture negative and 22 culture positive samples
- Individual milk samples (n = 114): 57 culture negative and 57 culture positive samples
- Bulk Tank Milk (BTM) samples (n = 221): not chosen based on culture results but rather by what was available in storage

All swab, semen and individual milk samples were collected from animals from dairy herds with a history of clinical mycoplasma-associated disease diagnosed by culture or PCR within the previous 2 years of sample collection. Of the 221 BTM samples, 215 were from dairy herds with a history of clinical mycoplasma-associated disease diagnosed by culture or PCR within the previous 2 years of sample collection, and 6 were from dairy herds with no clinical signs of mycoplasma-associated disease within the last 5 years of sample collection.

### Statistical analysis

Each bovine sample was classified as either positive or negative by culture and multiplex probe PCR. Samples were further classified as either ≥2 species identified or <2 species identified. Statistical analysis using a two-sample binomial test of proportions and the Kappa coefficient (Genstat 16^th^ Edition, VSN International, UK) was then performed separately for each classification method. The level of agreement between culture and PCR was calculated as the percentage of samples which had the same result for both tests. This was performed both individually for each sample type, as well as a whole on all samples. Statistical significance was declared at P<0.05.

## Results

### PCR specificity and limit of detection

Specificity results are shown in Table 2. All *M*. *bovis*, *M*. *californicum* and *M*. *bovigenitalium* isolates produced appropriate amplification. Amplification was not seen for any of the other Mollicutes or eubacterial isolates.

The limit of detection concentration (mean Ct ± SE) for each species in different sample types is shown in Table 3. For all three species, the limit of detection was best in milk samples, followed by semen samples and swab samples. Overall, the species *M*. *californicum* had the best limit of detection, followed by *M*. *bovis* and *M*. *bovigenitalium*. When all three species were present per sample, the limit of detection was poorer for all species in all sample types. In semen and swab samples, when all three species were present per sample, *M*. *bovigenitalium* was not detectable at the highest concentration.

**Table 3:** Multiplex probe PCR limit of detection (cfu/mL) and associated mean cycle threshold (±SE) for different spiked sample types. Single target species present per sample (A) and multiple target species present per sample (B) and the concentration of each target species in the sample at the limit of detection (read from left to right).

		Concentration of *Mycoplasma* species in sample (cfu/mL)		
A. Single species per sample		*M*. *bovis*	*M*. *californicum*	*M*. *bovigenitalium*^c^
Milk	*M*. *bovis* (C_T_ ± SE)	**1.3x10**^**2**^ **(35.0±0.1)**	0	0
	*M*. *californicum* (Ct ± SE)	0	**6x10**^**2**^ **(31.8±0.7)**	0
	*M*. *bovigenitalium* (C_T_ ± SE)	0	0	**5x10**^**5**^ **(28.8±0.2)**
Semen	*M*. *bovis* (Ct ± SE)	**1.3x10**^**5**^ **(30.8±0.7)**	0	0
	*M*. *californicum* (C_T_ ± SE)	0	**6x10**^**4**^ **(31.1±0.6)**	0
	*M*. *bovigenitalium* (C_T_ ± SE)	0	0	**1.4x10**^**7**^ **(24.6±0.7)**
Swabs	*M*. *bovis* (C_T_ ± SE)	**1.3x10**^**6**^ **(31.3±1.2)**	0	0
	*M*. *californicum* (C_T_ ± SE)	0	**6x10**^**4**^ **(33.5±1.9)**	0
	*M*. *bovigenitalium* (C_T_ ± SE)	0	0	**1.4x10**^**7**^ **(32.0±1.0)**
		Concentration of *Mycoplasma* species in sample (cfu/mL)		
B. Multiple species per sample		*M*. *bovis*	*M*. *californicum*	*M*. *bovigenitalium*^c^
Milk	*M*. *bovis* (C_T_ ± SE)	**1.3x10**^**5**^ **(30.2±0.03)**	6x10^3^	5x10^4^
	*M*. *californicum* (C_T_ ± SE)	1.3x10^6^	**6x10**^**4**^ **(29.9±0.9)**	5x10^5^
	*M*. *bovigenitalium*^a^ (C_T_ ± SE)	1.3x10^8^	6x10^6^	**5x10**^**7**^ **(34.7±2.0)**
Semen	*M*. *bovis* (C_T_ ± SE)	**1.3x10**^**7**^ **(27.7±1.1)**	6x10^5^	1.4x10^6^
	*M*. *californicum* (C_T_ ± SE)	1.3x10^7^	**6x10**^**5**^ **(33.0±0.6)**	1.4x10^6^
	*M*. *bovigenitalium*^b^ (C_T_ ± SE)	1.3x10^8^	6x10^6^	**1.4x10**^**7**^
Swabs	*M*. *bovis* (C_T_ ± SE)	**1.3x10**^**7**^ **(28.2±0.4)**	6x10^5^	1.4x10^6^
	*M*. *californicum* (C_T_ ± SE)	1.3x10^7^	**6x10**^**5**^ **(33.3±1.6)**	1.4x10^6^
	*M*. *bovigenitalium*^b^ (C_T_ ± SE)	1.3x10^8^	6x10^6^	**1.4x10**^**7**^

^a^ only seven out of nine replicates were positive across three PCR runs at the highest concentration tested

^b^ PCR could not detect *M*. *bovigenitalium* in semen or swabs when multiple *Mycoplasma* species were present. Values given are the highest concentrations tested.

^c^The concentration of *M*. *bovigenitalium* differs between spiked milk, and spiked semen and swabs due to a different culture with a different initial concentration being used in the different experiments.

### Bovine samples

Results for classification of samples as either negative or positive by culture and PCR are shown in Table 4. When analysing all bovine samples (n = 474), 27% (n = 130) were culture positive and 23% (n = 111) were PCR positive, with culture and PCR results in agreement for 90% (n = 425) of samples and having a Kappa coefficient of 0.73. No significant difference was observed between the proportion of culture positive and PCR positive samples (P = 0.156). Of the samples which were culture and PCR positive (n = 96), a disagreement in species identification was observed in seven samples (excluding multiple species identification). These included two swabs and five semen samples, all of which were identified as *M*. *bovigenitalium* by the culture method, but *M*. *californicum* by the multiplex probe PCR method.

**Table 4:** Comparison of culture and multiplex PCR for detecting bovine field samples as positive or negative for *M*. *bovis*, *M*. *californicum* or *M*. *bovigenitalium*.

bovine sample	Culture +	PCR +	P value	Level of Agreement^a^	Kappa
All (n = 474)	27% (n = 130)	23% (n = 111)	0.156	90% (n = 425)	0.73
swabs (n = 95)	49% (n = 47)	24% (n = 23)	<0.001	75% (n = 71)	0.49
semen (n = 44)	50% (n = 22)	50% (n = 22)	1.00	73% (n = 32)	0.46
individual milk (n = 114)	50% (n = 57)	48% (n = 55)	0.791	98% (n = 112)	0.97
BTM (n = 221)	2% (n = 4)	5% (n = 11)	0.066	95% (n = 210)	0.25

^a^ percentage of samples which had the same culture and PCR result.

Of the 95 swab samples, 49% (n = 47) were culture positive while only 24% (n = 23) were found to be PCR positive with a significant difference observed (P<0.001), a test result agreement of just 75% (n = 71) and a Kappa coefficient of 0.49. All 24 swab samples which were not in agreement were identified as *M*. *bovis* or *M*. *bovigenitalium* by culture, and negative by multiplex probe PCR. Of the culture positive swabs, 32% (n = 15) had less than six colonies of growth on agar (data not shown), none of which were detected as positive by PCR.

Of the 44 semen samples, 50% (n = 22) were culture positive and 50% (n = 22) were PCR positive, with no significant difference observed (P = 1.00), a test result agreement of 73% (n = 32) and a Kappa coefficient of 0.46. Of the 12 semen samples which were not in agreement, six were identified as *M*. *bovigenitalium* by culture but negative by multiplex probe PCR, and six were identified as negative by culture but *M*. *californicum*, *M*. *bovigenitalium*, or both by multiplex PCR.

For individual milk samples (n = 114), 50% (n = 57) were culture positive while 48% (n = 55) were PCR positive, with no significant difference observed (P = 0.791), a test result agreement of 98% (n = 112) and Kappa coefficient of 0.97. Both individual milk samples which were not in agreement were identified as *M*. *bovis* by culture and negative by multiplex probe PCR.

Analysis of BTM samples (n = 221) found only 2% (n = 4) of samples to be culture positive while 5% (n = 11) of samples were PCR positive, with a no significant difference observed (P = 0.066), a test result agreement of 95% (n = 209) and a Kappa coefficient of 0.25. Of the 11 BTM samples which were not in agreement, two were identified as *M*. *bovis* by culture and negative by multiplex probe PCR, and 9 were identified as negative by culture but *M*. *bovis* or *M*. *californicum* by multiplex probe PCR.

For the identification of multiple species per sample, when analysing all the sample types (n = 474) culture followed by 16S-23S rRNA sequencing of colonies did not identify any samples as having ≥2 species. However PCR did identify significantly more multiple species per samples with 1% (n = 5) of samples having two species present (P = 0.025; Kappa = 0). Three of these were from individual milk samples which all contained *M*. *bovis* and *M*. *californicum* as identified by the multiplex probe PCR. All three samples were culture positive for *M*. *bovis* only. The remaining two samples were from a swab (vaginal) and a semen sample which both contained *M*. *californicum* and *M*. *bovigenitalium* as identified by the multiplex probe PCR. The swab sample was culture positive for *M*. *bovigenitalium* only, while the semen sample was negative on culture.

## Discussion

The multiplex probe PCR limit of detection for *M*. *bovis*, *M*. *californicum* and *M*. *bovigenitalium* was investigated for milk, semen and swab samples. Only a single field isolate was used for each target species, and therefore the effect of inter-isolate variation was not assessed, however these results provide a guide to the expected limit of detection. Using the described extraction methods, the PCR limit of detection in milk for *M*. *bovis* and *M*. *californicum* was approximately 1.3x10^3^ cfu/mL and 6x10^2^ cfu/mL respectively. This is comparable to previous studies which have reported the limit of detection of *Mycoplasma* spp. in milk to be approximately 10^2^ cfu/mL following DNA extractions on inoculated milk samples, and analysis by probe based PCR [22, 23] and conventional endpoint PCR [29]. For *M*. *bovigenitalium* the limit of detection in milk at 5x10^5^ cfu/mL was higher than for the same previously published studies. However of the three target species, *M*. *bovigenitalium* is potentially of less concern in milk since its role in causing mastitis is debatable although this has not been fully explored. The average rate of shedding of *Mycoplasma* spp. from animals with clinical mastitis is 10^8^cfu/mL, and is reduced to10^6^cfu/mL or less in sub-clinically infected animals [27, 30]. As such, detection of clinically infected animals would be possible for all target species, with a strong possibility for the detection of many sub-clinical shedders.

When comparing culture and multiplex probe PCR, individual milk samples and BTM samples had the highest level of agreement of 98% and 95% respectively. BTM samples were the only sample type to have more samples identified as positive by multiplex probe PCR (n = 11) than by culture (n = 4), with the difference approaching significance (P = 0.066). This is an important finding given that BTM is the recommended sample type for biosecurity screening and monitoring *Mycoplasma* spp. activity at the herd level [17, 31], with several studies using BTM as a surveillance tool to identify herd level prevalence [21, 32].

The limit of detection and Ct values achieved at each dilution were higher in semen and swabs compared to milk for all three target species. For semen, this may be due to the complex nature of the sample type, which contains a very high level of DNA and protein, potassium ions, citric acid and fructose [33]. Bull semen samples also often contain environmental contamination, which can also inhibit the PCR reaction despite a thorough DNA extraction process [34]. This can cause a reduction in PCR efficiency, resulting in higher limits of detection and Ct values. Little work has been done on developing PCR assays to detect *Mycoplasma* species in bull semen, and the concentration of *Mycoplasma* species in naturally infected bull semen has not been investigated. Therefore comparisons between the limit of detection in semen cannot be made with previous studies. One study investigating the effects of inoculated bull semen on fertilization and embryo development suggested that 10^6^ and 10^4^ cfu/mL were high and low concentrations respectively for both *M*. *bovis* and *M*. *bovigenitalium* [35]. At these levels detection by the multiplex probe PCR would be possible for *M*. *bovis* and *M*. *californicum*, however questionable for *M*. *bovigenitalium*.

Previous studies have suggested bulls may play a role in *Mycoplasma* spp. dissemination via semen through both natural mating and artificial insemination [36, 37]. In vitro studies have demonstrated that *M*. *bovis* and *M*. *bovigenitalium* in inoculated semen can be transmitted and infect embryos via in vitro fertilization [35]. Associations have been demonstrated between the isolation of *M*. *bovis* and *M*. *bovigenitalium* from cows and reproductive disease including dystocia, endometritis and abortion [13, 38]. As such, identifying and developing the best method of detecting *Mycoplasma* spp. in semen is of value. When analysing bovine semen samples by culture and multiplex probe PCR, the level of agreement was only 73%, however equal numbers of samples were identified as positive by culture (n = 22) and PCR (n = 22). The multiplex probe PCR was unable to identify six culture positive samples, all of which were *M*. *bovigenitalium*. This may be due to the poorer limit of detection of *M*. *bovigenitalium* compared to the other target species. However the multiplex probe PCR was able to positively identify six samples which culture could not, all of which were *M*. *bovigenitalium* (n = 2), *M*. *californicum* (n = 3), or both (n = 1). The inability of culture to positively identify these semen samples may be due to sample storage or contamination which may have prevented the growth or identification of *Mycoplasma* spp. on agar; a recognized limitation of traditional culture. As all of these semen samples were *M*. *californicum* or *M*. *bovigenitalium*, an additional explanation could be that the agar media used may not support the growth of these species in a comparable manner to its support of *M*. *bovis* growth.

Swabs from mucosal surfaces can be used for identifying clinically and sub-clinically infected animals, with *Mycoplasma* spp. able to be isolated from the eye, nasal cavity, ear and vagina of dry and lactating cows, heifers and calves following an outbreak [39–41]. As such, swabs from mucosal surfaces may be a useful sample type when screening for *Mycoplasma* spp. in the event that milk samples are not available (e.g. non lactating stock). In this study, the limit of detection from swabs (vaginal, preputial, nose and eye) was higher than milk and semen for all three species, however it was representative of the bacterial concentration in the solution in which the swabs were dipped into. As swabs only hold approximately 10 μL of liquid, the actual amount of organism present on the swab is likely to be at least 100 fold less than the concentration of the solution. Therefore the limit of detection is comparable with previous studies which could detect mycoplasma in spiked nasal swabs down to 2x10^3^ cfu [42].

When comparing culture and multiplex probe PCR, significantly more swab samples were identified as positive by culture (n = 47) than by multiplex probe PCR (n = 23) with an agreement of 75%. However this may have been due to the sample analysis procedure itself with all swabs first being inoculated onto Mycoplasma agar before PCR extractions were performed. Consequently the inoculation process would have removed some DNA from the swab, with less available for the extraction process. This theory is supported by the fact that 32% of the culture positive swabs grew less than six *Mycoplasma* spp. colonies on agar. While duplicate swabs samples could have been taken to help avoid this limitation, it could not be ensured that equal quantities of organism would be present on duplicate swabs in order to make an equal comparison. In a diagnostic situation, it is unlikely that both culture and DNA extraction followed by PCR would be performed on the same sample, but rather one method employed and so loss of DNA would not occur from the swab prior to processing to the same extent as occurred here.

When all three target species were present within a sample, the limit of detection increased by 10 to 1000 fold, and the Ct values achieved at each dilution increased, for all species in all sample types. This may be due to competition for reagents to amplify multiple species within the PCR, causing a reduction in the PCR efficiency. The degree of change in the limit of detection may also be dependent on the concentrations of *M*. *bovis*, *M*. *californicum* and *M*. *bovigenitalium* in the sample, which was not fully investigated. For *M*. *bovigenitalium*, detection was not possible in semen and swab samples when *M*. *bovis* and *M*. *californicum* were also present in high concentrations and this would need to be taken into consideration when interpreting results. However, this also may be dependent on the concentration of *M*. *bovis* or *M*. *californicum* in the sample, as several swab samples were identified as containing *M*. *bovigenitalium* and *M*. *californicum* by multiplex probe PCR. Therefore while a multiplex probe PCR may allow benefits of identifying three *Mycoplasma* species in the one reaction, greater efficiency and a reduction in reagent use, the limit of detection may be sacrificed if all three species are present in the one sample. Previous studies have identified the presence of two *Mycoplasma* species in single bulk tank milk samples [21, 24], however greater than three species in a single sample is rarely reported. Therefore this limitation may not prove to be an issue.

Of the samples analysed, culture followed by 16S-23S rRNA sequencing of colonies was not able to identify more than one species present from agar growth, while the multiplex probe PCR was able to identify five samples as having two species. All five samples were identified by multiplex probe PCR as both *M*. *bovis* and *M*. *californicum* (n = 3), or *M*. *californicum* and *M*. *bovigenitalium* (n = 2). The inability of culture to identify more than one species may be due to the speciation technique, which involved several colonies of growth being selected for 16S-23S rRNA PCR and sequencing from among potentially many colonies on a plate. This could have resulted in one of the species not being selected. However, it is also possible that one of the species did not grow on the plate despite being present in the sample due to overgrowth by more competent, faster growing or more numerous *Mycoplasma* species, or due to variations in media suitability between species. By performing extractions on the original sample followed by multiplex probe PCR, there may be a greater representation of the actual species present in the sample, without inadvertently selecting for certain species that can occur via culture.

## Conclusion

This study evaluated the analytical performance of a single multiplex probe PCR assay against traditional culture for the detection of *M*. *bovis*, *M*. *californicum* and *M*. *bovigenitalium* in bovine milk, semen and swab samples. For multiplex probe PCR specificity, all *M*. *bovis*, *M*. *californicum* and *M*. *bovigenitalium* isolates produced appropriate amplification. Amplification was not seen for any of the other Mollicutes or eubacterial isolates. The limit of detection for the multiplex probe PCR was best in spiked milk samples, followed by semen and swab samples. When all three *Mycoplasma* species were present in spiked samples, the limit of detection increased by 10 to 1000 fold for all species in all sample types. *M*. *bovigenitalium* had the poorest limit of detection for all sample types and was not able to be identified in semen or swab samples when all three *Mycoplasma* species were present. Overall, when comparing culture and multiplex probe PCR, there was no significant difference in the proportion of culture and PCR positive field samples for all sample types. However individually, culture could detect significantly more positive swab samples. No significant differences were identified for semen, individual milk samples or BTM samples. For the identification of multiple species per sample, multiplex probe PCR identified five samples with two species present however the culture method did not enable identification of more than one species. Therefore the method of choice for identification of *M*. *bovis*, *M*. *californicum* and *M*. *bovigenitalium* in bovine samples may involve consideration of the sample type being analysed, and whether the identification of multiple target species is required.

## Supporting information

S1 File Multiplex Probe PCR Limit of Detection Data for Spiked Milk. (XLSX)

S2 File Multiplex Probe PCR Limit of Detection Data for Spiked Semen. (XLSX)

S3 File Multiplex Probe PCR Limit of Detection Data for Spiked Swabs. (XLSX)

S4 File Culture and Multiplex Probe PCR Results for Bovine Field Samples. (XLSX)
